# Probiotic and postbiotic analytical methods: a perspective of available enumeration techniques

**DOI:** 10.3389/fmicb.2023.1304621

**Published:** 2023-12-07

**Authors:** Marie-Eve Boyte, Andrzej Benkowski, Marco Pane, Hanan R. Shehata

**Affiliations:** ^1^NutraPharma Consulting Services Inc., Sainte-Anne-des-Plaines, QC, Canada; ^2^Eurofins, Madison, WI, United States; ^3^Probiotical Research s.r.l., Novara, Italy; ^4^Purity-IQ Inc., Guelph, ON, Canada

**Keywords:** digital PCR, real-time PCR, enumeration, quantification, viable count, flow cytometry, plate count, culture-independent

## Abstract

Probiotics are the largest non-herbal/traditional dietary supplements category worldwide. To be effective, a probiotic strain must be delivered viable at an adequate dose proven to deliver a health benefit. The objective of this article is to provide an overview of the various technologies available for probiotic enumeration, including a general description of each technology, their advantages and limitations, and their potential for the future of the probiotics industry. The current “gold standard” for analytical quantification of probiotics in the probiotic industry is the Plate Count method (PC). PC measures the bacterial cell’s ability to proliferate into detectable colonies, thus PC relies on cultivability as a measure of viability. Although viability has widely been measured by cultivability, there has been agreement that the definition of viability is not limited to cultivability. For example, bacterial cells may exist in a state known as viable but not culturable (VBNC) where the cells lose cultivability but can maintain some of the characteristics of viable cells as well as probiotic properties. This led to questioning the association between viability and cultivability and the accuracy of PC in enumerating all the viable cells in probiotic products. PC has always been an estimate of the number of viable cells and not a true cell count. Additionally, newer probiotic categories such as Next Generation Probiotics (NGPs) are difficult to culture in routine laboratories as NGPs are often strict anaerobes with extreme sensitivity to atmospheric oxygen. Thus, accurate quantification using culture-based techniques will be complicated. Another emerging category of biotics is postbiotics, which are inanimate microorganisms, also often referred to as tyndallized or heat-killed bacteria. Obviously, culture dependent methods are not suitable for these products, and alternative methods are needed for their quantification. Different methodologies provide a more complete picture of a heterogeneous bacterial population versus PC focusing exclusively on the eventual multiplication of the cells. Alternative culture-independent techniques including real-time PCR, digital PCR and flow cytometry are discussed. These methods can measure viability beyond cultivability (i.e., by measuring cellular enzymatic activity, membrane integrity or membrane potential), and depending on how they are designed they can achieve strain-specific enumeration.

## Introduction

Probiotics, which represent the largest category of non-herbal/traditional dietary supplements worldwide, are experiencing significant growth. The global market size for probiotics was valued at USD 58.17 billion in 2021 and is anticipated to grow at a compound annual growth rate (CAGR) of 7.5% from 2021 to 2030 (Grand-View-Research-Inc, 2022).

The World Health Organization in 2002 initially defined probiotics as “live microorganisms which when administered in adequate amounts confer a health benefit on the host” (FAO/WHO, 2002). The definition was later refined in 2014 to “live microorganisms that, when administered in adequate amounts, confer a health benefit on the host” (Hill et al., 2014), a statement that has gained broad acceptance within both the scientific community and the industry. According to this definition, a probiotic strain must be viable in an appropriate quantity to confer a health benefit to the consumer. However, this definition does not provide any specific standards to identify or quantify this viability, but the common practice is to measure viability using direct plate count (PC) enumeration which expresses results in Colony Forming Units (CFUs).

Breeuwer and Abee in 2000 proposed a broader definition of bacterial viability as having an “intact cytoplasmic membrane, protein and other cell components synthesis (nucleic acids, polysaccharides, etc.) and energy production necessary to maintain cells metabolism; and, eventually, growth and multiplication” (Breeuwer and Abee, 2000). Building on Breeuwer and Abee’s definition, a variety of methodologies can provide a more comprehensive view of the viability of a heterogeneous bacterial population than the traditional PC method, which focuses solely on growth and multiplication potential of a subset of the bacterial population. Moreover, the emergence of a new generation of probiotics comprising strictly anaerobic bacteria presents significant challenges for enumeration using traditional PC methods, making it necessary to explore alternative techniques that can assess their viability and provide a more accurate cell count.

This paper will delve into the most widely used methods for quantifying and assessing the viability of probiotic strains, discuss their limitations, and explore alternative techniques that overcome these challenges. The paper will also introduce the concepts and applications of culture-dependent and culture-independent enumeration methods. To provide a general overview of the status of viability acceptance across different regulations and guidelines we did provide a summarized table as a reference (Table 1).

**Table 1:** Overview of the status of viability acceptance across different regulations and guidelines.

No.	Country	Comments	References
1	Italy	The recommended product serving for daily consumption shall contain a quantity of 10^9^ live cells of at least one of the strains. It is pointed out that the most suitable analysis methods to quantify live micro-organisms may vary according to each species.	Ministero-Della-Salute-Italy (2018)
2	France	The recommended product serving for daily consumption shall be between 10^7^ and 10^9^ viable cell per day from one strain.	DGCCRF (2023)
3	Australia	The quantity (potency) of each strain must be expressed in CFU/g, CFU/mL or CFU per metric unit or dose; or as the number of viable cells per mL based on a viable-cell assay.	TGA (2023)
4	Europe	For live biotherapeutics, the potency of each strain expressed in CFU/mL, CFU/g, CFU/unit or viable cells/mL. For food or food supplements, there is no specific legislation that regulates the use of probiotics in human nutrition, therefore the EU legislation does not specify any specific labeling provisions for probiotic enumeration reporting other than for the approved claim which must be reported in CFU.	EDQM (2019) and IPA (2022)
5	Codex alimentarius	From a labeling side, the product label should contain the amount of viable cells of total probiotic microorganisms (CFU/g). Although, from an enumeration side, traditionally, plating has been used and endorsed as the “standard way” to evaluate microbial viability and it has been determined through counting “colony-forming units,” CFU. The plate count method is based on the premise that a single bacterium can grow and divide to give an entire colony. This method is historically and currently, the most broadly used method to demonstrate the activity of the microorganisms. Now, other methods such as flow cytometry (ISO 19344 IDF 232) are coming to be used widely and a standardized method has been developed and used as a way to evaluate total probiotic microorganisms. All work will be coordinated with the applicable general subject Codex Committee to ensure the appropriate application of Codex. Expertise and resources.	CCNFSDU (2019)
6	Norway	The number of viable probiotic bacterial cells in the product within the time frame of its shelf life should be clearly given including a proviso that recommended storage conditions have been upheld. The numbers may be expressed as log Colony Forming Units (CFU) per gram of product or per serving of a specified size.	Yazdankhah et al. (2014)
7	USA	For dietary supplements, it is mandatory to declare the quantitative amount of live microbial ingredients in terms of weight in the Supplement Facts label. The concentration can be declared in CFU as long as it is done in a manner that clearly separates and readily distinguishable from the weight. However, the FDA believes that CFUs provide a useful description of the quantity of live microbial dietary ingredients and is aware that researchers are currently evaluating other methods and units of measure for live microbial dietary ingredients and that such alternative methods have the potential to more accurately and more efficiently quantify the number of viable cells. For food containing microorganisms, such as yogurt, the product label may be indicate “contains live and active cultures” or another appropriate descriptor if the food contains a minimum level of live and active cultures of 10^7^ colony forming units per gram (CFU/g) at the time of manufacture with a reasonable expectation of 10^6^ CFU/g through the manufacturer’s assigned shelf life of the product.	FDA (2018) and FDA (1977)
8	Brazil	The product must be labeled with the quantity to be consumed in CFU/day to obtain the desired effect.	ANVISA (2021)
9	India	Minimum viable number of added probiotic organisms in food shall be ≥10^8^ CFU in the recommended serving size per day.	FSSAI (2022)
10	Canada	All individual strain quantities of live microorganisms must be indicated in Colony Forming Units (CFU) per dosage unit.	Health-Canada (2023)
11	Colombia	The food should contain a number of viable cells ≥1 × 10^6^ CFU/g in the finished product until end of shelf life	Ministry-of-Health-and-Social-Protection-of-Colombia (2011)
12	IPA	The quantitative amount(s) of probiotics in a product should be expressed in Colony Forming Units (CFUs).	CRN-IPA (2017)

## Culture-dependent enumeration methodologies

The traditional microbiological PC method is the most common choice for enumerating viable beneficial microorganisms and contaminants in international standards. These standards are issued by bodies such as the International Organization for Standardization (ISO), the International Dairy Federation (IDF), Bacteriological Analytical Manual (BAM), and the United States Pharmacopeia (USP). The PC method measures the ability of bacterial cells to proliferate into detectable colonies on agar media, presenting results in Colony Forming Units (CFUs). The Colony Forming Unit (CFU) has been the unit for microbial enumeration for at least 125 years (USP, 2018). This method’s popularity arises from its technical simplicity, ease of implementation, and wide acceptance, marking it the ‘gold standard’ in the probiotic industry for the analytical quantification of probiotics (Weitzel et al., 2021). The PC method, however, has multiple disadvantages such as laborious workload and lengthy periods of incubation (USP, 2019). Additionally, it should be noted that a CFU count has always been an estimation of the number of viable microorganisms present and not a true cell count (Davey, 2011; USP, 2018). The viable counts estimated using culture-dependent methods rely on the suitability of the growth media and incubation conditions for the strain to be quantified (Wendel, 2022). Furthermore, the applied method will likely change the qualitative and/or quantitative properties of the original sample since the selective pressure may alter its native composition and state. This is specifically true for probiotic blends where the additional variable of the interaction between strains during the incubation time can shift the relative abundances of the original sample (Sielatycka et al., 2021).

The variability between species and between strains in response to plating procedures also means that no single methodology can be universally applied to all probiotic organisms (Davis, 2014). This complexity extends to enumerating species or strains in a complex blend. In response, probiotic strain manufacturers have developed PC methods that utilize chemical components to promote or inhibit growth of specific bacterial taxa (Davis, 2014). For example, MRS (deMan Rogosa, Sharpe) agar is commonly used for *Lactobacilli* enumeration (Champagne et al., 2011). However, when supplemented with raffinose and lithium chloride, it enables the growth of *Bifidobacteria* (Hartemink et al., 1996). Another example is adding 0.5 ppm of clindamycin to MRS medium to allow the enumeration of heterofermentative *Lactobacillus* genus (Van de Casteele et al., 2006; Davis, 2014). It is well recognized that the high number of variables that can affect PC enumeration generates a continuous debate on which methodology to correctly apply. Recently, the USP probiotic panel working group published a comprehensive overview of the Analytical Procedure Lifecycle Management (APLM) for comparing PC methods. This approach is universal as it is a process to define procedure performance based on the concept that the reportable value must fit its intended use; therefore, information gathered through APLM can be used to evaluate and compare any procedure (Weitzel et al., 2021).

The emergence of novel dosage forms of probiotics, such as gummies and oils, and their blending with other active ingredients like herbs, fruits and vegetable extracts, vitamins, and minerals, adds another layer of complexity when using PC methods or any alternative enumeration method. For example, bacteria can remain trapped within gummy particles, resulting in underestimation of the total count, or the cell growth in culture media may be inhibited by other ingredients in the products. Consequently, with every new active ingredient and delivery form, testing laboratories need to validate the method to ensure scientific validity and fitness for purpose, thereby requiring additional financial investment, time, and human resources.

Given the numerous variables that can affect PC enumeration, the industry has accepted a variability range between 20–30% or a Relative Standard Deviation (RSD) of 10–15% (Hansen et al., 2018). The Italian Ministry of Health and the European Scientific League for Probiotics (ESLP) have also provided guidelines and, the latter, quality seals based on scientific evaluation and control of the CFU content, respectively with a variability of 0.5 and 1 log at the end of the product shelf-life (Warzée, 2016). Despite these efforts, the question remains as to the best methodology for microbial enumeration, given the high variability and lack of precision inherent in PC methods. The challenges associated with this evaluation highlight the need for both standard PC enumeration methods and alternative techniques to ensure accurate quantification and enumeration of probiotics.

In addition to technical difficulties in enumerating probiotics belonging to traditional probiotic taxa like *Bifidobacterium* spp. and, *Lactobacillus* spp., the industry is confronted with additional challenges when enumerating novel microorganisms, often referred to as Next-Generation Probiotics (NGPs) (O’Toole et al., 2017; Saarela, 2019; Singh and Natraj, 2021; Torp et al., 2022). NGPs are “live microorganisms identified on the basis of comparative microbiota analyses that, when administered in adequate amounts, confer a health benefit on the host” (Martín and Langella, 2019). An alternate term that is proposed for NGP is Live Biotherapeutic Product (LBP) (Martín and Langella, 2019). Many of these organisms, such as *Akkermansia muciniphila*, *Faecalibacterium prausnitzii*, *Eubacterium hallii*, *Prevotella copri*, *Bacteroides* spp., *Roseburia* spp. (Meehan and Beiko, 2014), *Bacteroides uniformis* (Gomez-Arango et al., 2016), *Christensenella minuta* (Goodrich et al., *2014*), *Oxalobacter formigenes* (Stewart et al., 2004), and *Alistipes putredinis* (Png et al., 2010), are highly adapted to the gastrointestinal environment or other human body niches. These NGPs are often strict anaerobes, highly sensitive to atmospheric oxygen, thus necessitating specific growth conditions and advanced culturing techniques to grow them in a laboratory setting (O’Toole et al., 2017; Saarela, 2019; Singh and Natraj, 2021; Torp et al., 2022). Achieving appropriate growth conditions that mimic their native environments is far from a trivial task and often involves intricate adjustments (O’Toole et al., 2017; Saarela, 2019). Thus, quantification of these NGPs using traditional culture-based techniques proves complex, and the use of culture-independent methods becomes highly advantageous as they can provide a more accurate assessment of viability, addressing a critical need where traditional culture-based methods may fall short (Chang et al., 2019; Saarela, 2019; Singh and Natraj, 2021; De Filippis et al., 2022; Torp et al., 2022).

## Importance of strain specificity

The concept of bacterial strain identity has undergone considerable transformation with the advent and progression of molecular methodologies that offer precise and distinct identification of bacterial genomes. Traditionally, bacterial strains have been identified through laborious culture-based methods, with the definition rooted in taxonomic practices and phenotypic traits.

According to the first edition of Bergey’s Manual of Systematic Bacteriology, ‘a strain is made up of the descendants of a single isolation in pure culture and usually made up of a succession of cultures ultimately derived from an initial single colony’ (Staley and Krieg, 1984). This definition inherently ties a bacterial strain to the process of *in vitro* culturing and isolation of a bacterial colony. This implies that the existence of a strain, as defined within the scientific context, is tied to the human act of isolation, and not as a natural entity within the ecosystem it was derived from Achtman and Wagner (2008).

However, the narrative has gradually evolved, largely owing to advancements in genomic technology. The strain, as we refer to it in the current context, is often more closely associated with a human-operated setting, an artifact of the laboratory environment and techniques used to isolate and culture it, rather than a naturally occurring, distinct entity within its ecological niche (Gevers et al., 2005). According to Thea Van Rossum et al., 2020, the biological basis for strain definition is not well established and may not exist (Van Rossum et al., 2020).

This shift in perspective opens up important dialogs on the biological relevance and ecological roles of bacterial strains as we have defined them (Doolittle and Papke, 2006). It also underscores the potential discrepancies that may arise when translating laboratory findings to a more complex, real-world context (Polz et al., 2013). Given these considerations, it becomes increasingly important to re-evaluate and contextualize the concept of strains within the broader framework of bacterial ecology and evolution. This is an area where continued advancements in genomics and related fields can contribute significantly to our understanding of microbial diversity and function (Koeppel and Wu, 2013).

A modern definition by Ghazi et al. (2022) proposes a strain as “a collection of cells or genomes within a relatively small range of phylogenetic variation (i.e., a very narrow subspecies clade).” With species identity often defined by approximately 95–97% of whole-genome nucleotide sequence similarity, a strain could represent even greater sequence similarity, up to >99% or > 99.9% whole-genome sequence similarity. Theoretically, even one single nucleotide polymorphism (SNP) could delineate strain identity, although no concrete rules have been established on how many SNPs define a unique strain or whether such SNPs need to result in phenotypic changes to justify strain discrimination (Ghazi et al., 2022). This leads to the consideration that SNPs alone may not be sufficient for strain discrimination and suggests the need to employ multiple methodologies to fully comprehend a strain’s uniqueness, also including factors such as clinical and intellectual property backgrounds of the strain.

The concept of strain-specificity in probiotics has traditionally been considered the cornerstone of probiotic science. To meet the World Health Organization’s definition of probiotics, a probiotic microorganism must exhibit a health benefit, and any claims of a specific health benefit must be supported by strain-level clinical evidence. It is generally accepted that a probiotic’s beneficial effects on the host will be specific to a particular strain, and that the characteristics and efficacy of a certain strain cannot be generalized to other strains within the same species, or to strains of other species (Lee et al., 2013). A systematic review of the literature and various meta-analyses conducted in 2018 suggests that there is strong evidence showing that the efficacy of probiotics is both strain-specific and condition-specific (McFarland et al., 2018). The strain specificity of probiotic health benefits highlights the importance of methods that enable strain-specific identification and enumeration of probiotics in both research and production settings to confirm product efficacy.

While culture-dependent PC methods and their corresponding CFU counts are still considered the gold standard for quantification of probiotic bacteria, they lack the specificity required to quantify individual strains in a multi-strain blended material. Therefore companies will often rely on a combination of assays to confirm both identity and quantity as respective datasets. It usually involves a total count of CFUs present or a quantification to the genus-level and a separate confirmation of identity using a genomic application as described above often at species level resolution (Jackson et al., 2019).

Alternatively, a company may rely on raw material concentration information and formulation targets to determine a theoretical number of probiotic bacteria present in the finished product (Quantification by Input); but this approach lacks the confirmation of cellular viability in the final product as ingredients are subjected to manufacturing processes and potentially negative interactions with other active ingredients. Since strain-level quantification in a blend cannot be achieved using traditional PC techniques, methods based on real-time quantitative PCR (rtPCR or qPCR), digital chip-based or droplet PCR (cdPCR or ddPCR) (Hansen et al., 2018; Shehata et al., 2023), or antibody-coupled flow cytometry (Chiron et al., 2018) have been developed to combine identification with quantification to enumerate probiotics at the strain level within a coherent methodological validation setting. Keep into account that the concept of strain specificity is fluid and that if product design implies one micro-organism or different species or genus (and not different strains of the same species) any methodologies that discriminate at the species and genus level shall be considered valid, especially with the broader definition of the strains as the sum of the genetic, phenotypic, productive, pre-clinical, clinical and intellectual proprieties evidences.

## Alternatives in viability definition

Although the concept of viability was primarily gauged by cultivability, i.e., the ability of a cell to replicate and form a colony on agar media (USP, 2018; Fiore et al., 2020), there was agreement that the definition of viability should not be constrained to cultivability alone (Wendel, 2022). For instance, bacterial cells may exist in a viable but not culturable (VBNC) state, where cells lose the ability to form colonies – yet maintain membrane integrity, enzyme activity, a pH gradient, and high levels of rRNA (Lahtinen et al., 2006b, 2008; Fiore et al., 2020; Wendel, 2022) – as a survival strategy for microorganisms under various environmental stresses (İzgörd et al., 2022). This distinguishes VBNC cells from dead cells, which exhibit irreversibly damaged cell membranes and no metabolic activity (Li et al., 2014).

The concept of Viable But Non-Culturable (VBNC) cells has garnered increasing attention in the realm of probiotic enumeration as well as in the broader context of microbial ecology. Traditional methods like Plate Count (PC) often underestimate the actual number of viable cells, as they do not account for cells in the VBNC state. These cells, although not cultivable can exhibit probiotic properties (Kiepś et al., 2023).

While VBNC cells cannot grow and form colonies on agar without resuscitation, they are nonetheless viable (Davis, 2014), thus challenging the conventional association between viability and cultivability (Wendel, 2022). This discrepancy has led to scrutiny of the accuracy of culture-dependent enumeration methods for evaluating all viable cells in probiotic products (Foglia et al., 2020; Visciglia et al., 2022). Consequently, a cell count obtained through culture-dependent methods is now considered an estimate rather than an accurate viable cell count (USP, 2018). This is because PC methods may potentially underestimate viable cell numbers, as they fail to detect VBNC cells (Jackson et al., 2019; Fusco et al., 2022). Recent advancements in enumeration techniques, such as Imaging Flow Cytometry (IFC),staining-based flow cytometry and viability qPCR, have shown promise in capturing the VBNC population more accurately (Ma et al., 2023; Pereira et al., 2023; Shehata et al., 2023).

Interestingly, VBNC probiotic populations may contribute to health benefits within the host (Wendel, 2022), as VBNC cells can resuscitate, regain the ability to divide, and interact with the host upon encountering favorable conditions in the gut (Pinto et al., 2015; Fiore et al., 2020; Puntillo et al., 2022). This phenomenon mirrors that of pathogenic bacteria in a VBNC state (Li et al., 2014; Zhao et al., 2021, 2022), which have been found to regain pathogenicity and virulence after resuscitation (Li et al., 2014). Resuscitation from the VBNC state has been widely studied, especially for risk control of recovered pathogenic or spoilage bacteria. The phenomenon of resuscitation is crucial for proving the existence of the VBNC state and has potential applications in the food industry (Pan and Ren, 2022). One of the major advances in resuscitating VBNC cells is the discovery of bacterial cytokine proteins like resuscitation-promoting factor (Rpf), which have potential applications in environmental bioremediation (Xie et al., 2021). Moreover, short-chain fatty acids (SCFAs) have been identified as potential resuscitation factors that can break the dormancy of certain marine bacteria within 5 days (Sun et al., 2023). Metabolomic studies have revealed significant differences between VBNC and recovered cells, particularly in *Lacticaseibacillus paracasei* Zhang, a probiotic and starter strain. Levels of specific amino acids like L-cysteine, L-alanine, L-lysine, and L-arginine notably increased in revived cells, suggesting altered physiology in the VBNC state (Wang et al., 2023).

This has led to requests to extend the probiotic viability definition beyond cultivability to probiotic activity, which can be measured based on membrane integrity, metabolic activity, membrane potential, or RNA content (Davey, 2011).

Understanding the physiology and metabolism of VBNC cells is essential for both risk control and the exploration of beneficial microbial resources (Pan and Ren, 2022).

**Figure 1:**
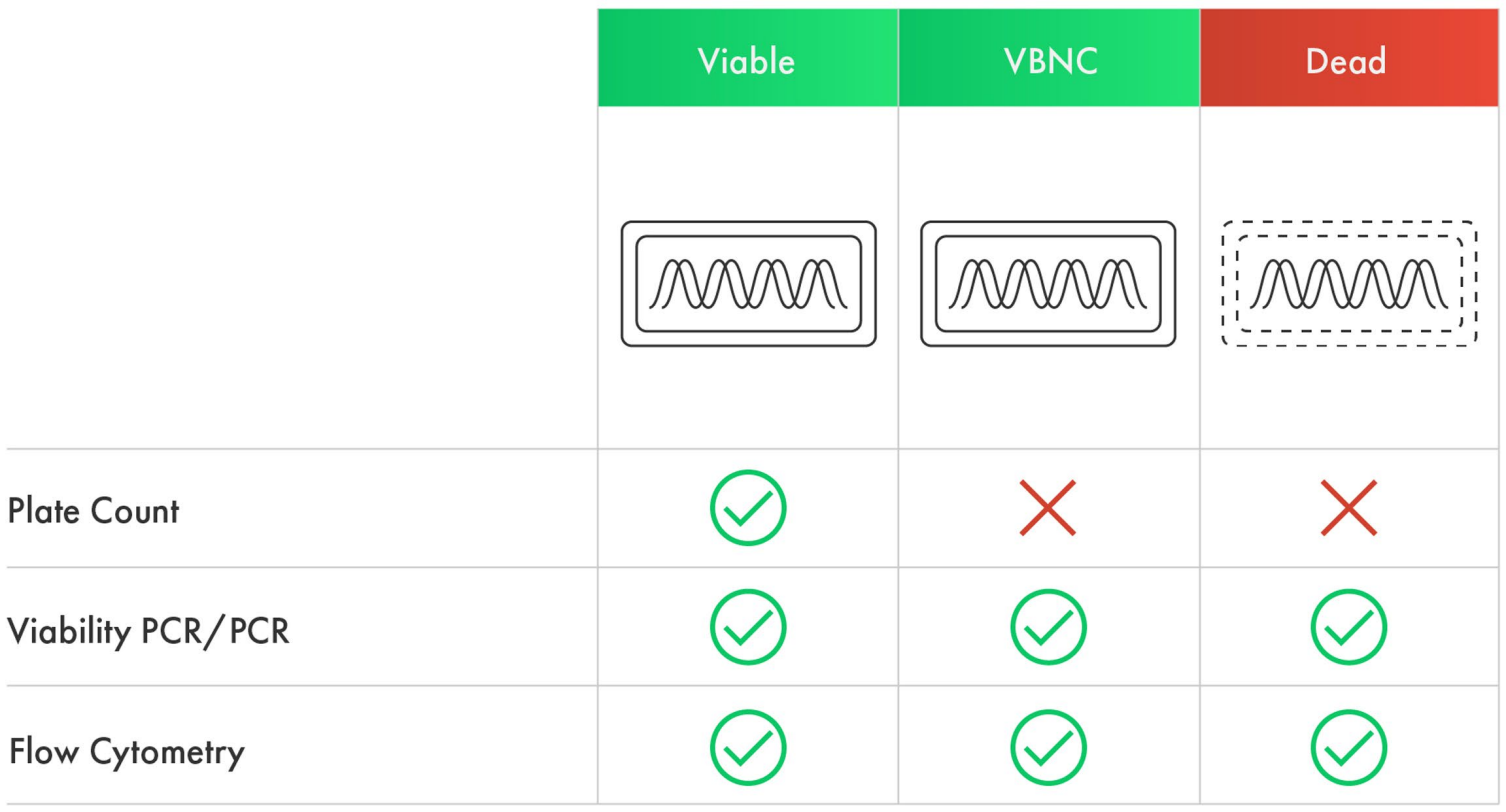
The ability of culture-dependent and culture-independent methods to detect viable, VBNC, and dead cells.

Given the potential role VBNC cells may play within the host, it is crucial to consider enumeration methods that account for cells in this state. Culture-independent methods could potentially count both culturable and VBNC cells, yielding more accurate viable counts (Figure 1). This is particularly important for finished probiotic products and during shelf life, as probiotic cells undergo a dynamic shift to enter a VBNC state during shelf life and upon exposure to stresses during storage (Davis, 2014; Foglia et al., 2020). This shift to a VBNC state results in a disparity between CFU counts and actual viable counts (Wendel, 2022), thus it has been observed as a gap between counts determined using culture-dependent and culture-independent methods (Foglia et al., 2020; Visciglia et al., 2022; Shehata et al., 2023).

Unlike VBNC cells, dead cells that have the capability to interact with the host eliciting a potential health benefit do not qualify as probiotics according to the WHO definition (Binda et al., 2020). They are instead referred to as “postbiotics” (Aguilar-Toalá et al., 2018).The emerging category of postbiotics refers to a preparation of inanimate microorganisms and/or their components that confer a health benefit on the host (Salminen et al., 2021). These inanimate microorganisms, often referred to as tyndallized or heat-killed bacteria, need to be characterized before inactivation (Salminen et al., 2021). There are many inactivation methods, but currently heat treatment is the preferred method in the industry and the most historical (Piqué et al., 2019; Rabiei et al., 2019; Vallejo-Cordoba et al., 2020). Interest in postbiotics is increasing due to factors such as their higher stability during industrial preparation, longer shelf life compared to probiotics, ease of transport and storage, and compatibility with products where viability is a challenging parameter (Salminen et al., 2021). However, this class of products cannot be enumerated by culture-dependent methods, and alternative quantification methods are needed. A bacterial counting chamber could be used, where cells are treated with dyes like propidium iodide that stain bacteria with damaged membranes (dead bacteria) only (Lahtinen et al., 2006b; Sugahara et al., 2017). Culture-independent methods would also be useful for enumerating postbiotics. For instance, the recent approval of *Akkermansia municiphila* as a Novel Food in Europe (Turck et al., 2021) pursuant to Regulation (EU) 2015/2283, is a notable example, where the dose was enumerated in Total Fluorescent Units (TFU) by flow cytometry with a safety target of <10 CFU/g.

## Flow cytometry (FCM), a modern method to measure different viability parameters

Flow cytometry has emerged as an advanced tool in probiotic viability assessment, capable of extracting detailed information on individual cells including their size, granularity, and morphology through the analysis of laser light scattering. This technique leverages the ISO 19344 IDF 232 lactic acid bacteria enumeration method, utilizing three different staining protocols to evaluate cellular enzymatic activity, membrane integrity, and membrane potential, providing comprehensive insights into bacterial viability (ISO, 2015).

**Figure 2:**
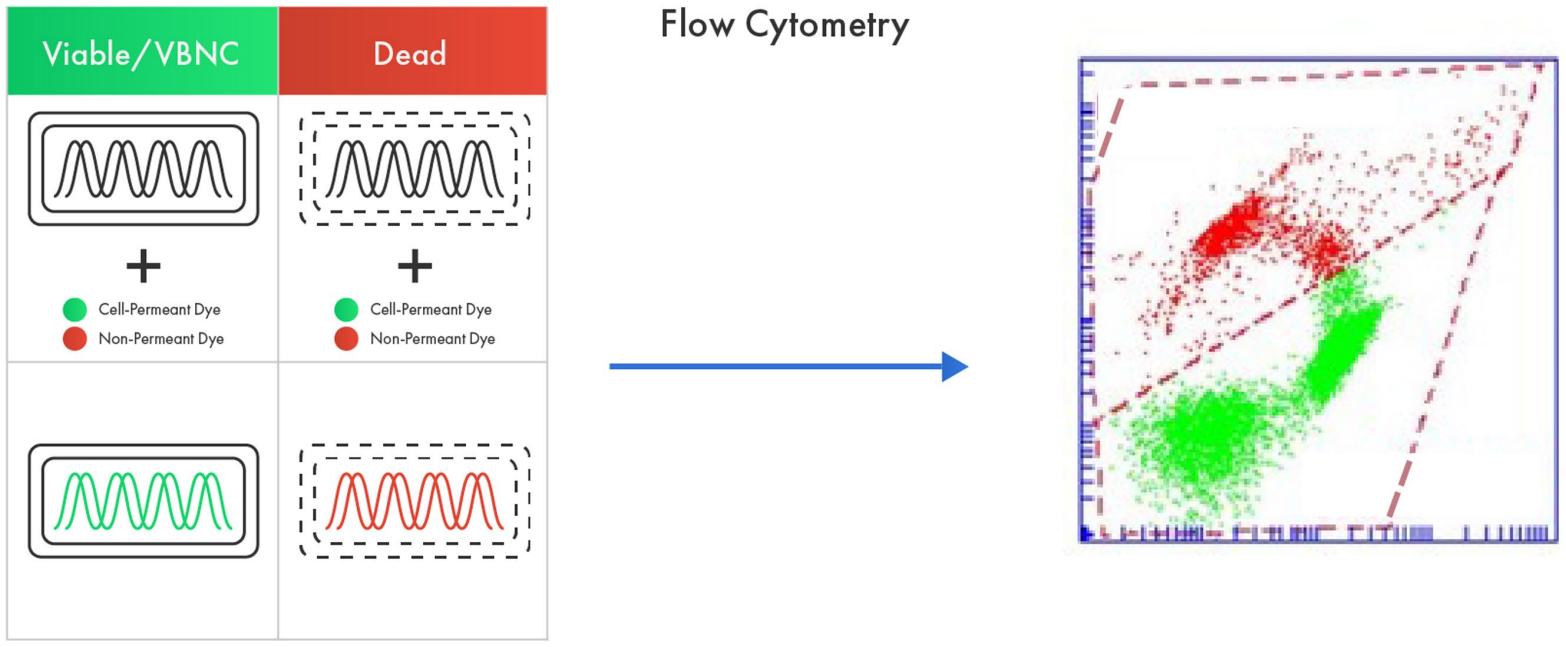
Flow cytometry as an advanced tool in probiotic viability assessment based on membrane integrity. The method utilizes a cell permeant dye that penetrates all bacterial cells regardless of their viability (live, damaged, and dead) and a non-permeant dye that only enters dead bacterial cells. The difference between the fluorescence from two dyes represents viable cells.

The membrane integrity protocol for example, employs a DNA binder colorant that penetrates all bacterial cells (SYTO 24) regardless of their viability (thereby identifying Total Fluorescent Units, TFU: bacteria that are alive, damaged, and dead) and another colorant which only enters bacterial cells with a compromised membrane (Propidium Iodide) (Figure 2). The difference between the two groups is expressed as Active Fluorescent Units (AFU) which represents the viable cells (cells with intact membranes) based on this protocol. Total Fluorescent Units (TFU) provide information on the total number of cells in the sample, whereas the difference between TFU and AFU (TFU-AFU), termed as non-AFUs (n-AFUs), represents the dead, likely irreparable, bacterial population (Fallico et al., 2020; Ma et al., 2023). The enzymatic activity protocol is based on fluorescence generated by the non-fluorescent dye Carboxylfluorescein diacetate succinimidyl ester (cFDA) when it is cleaved by cellular esterase (a proxy of cellular viability), meanwhile the membrane potential is based on DiOC_2_ that binds the membrane with a green fluorescence emission; when cells are activated the maximum fluorescence is then red-shifted.

Apart from the fluorescence techniques, ISO 19344 has been validated using a broad array of probiotic species, including *Lactobacillus* spp., *Bidifobacterium* spp., and *Streptococcus* spp., which emphasizes the method’s capability to be utilized for enumerating any strain belonging to the validated species, thereby offering a generalized approach which overcomes the intrinsic limitations of cultivability methods that rely on specific protocols that can vary according to the taxonomical species under examination. These technical advantages make FCM a more accurate and faster technique compared to PC enumeration, as FCM directly enumerates each single cell in a given sample and provides information on the bacterial population heterogeneity based on the fluorochrome used (Sielatycka et al., 2021; Ma et al., 2023).

It is important to note, ISO 19344 has been validated on fresh samples, and no data have been provided on aging samples and/or stability data. It is generally accepted that AFU and CFU data tend to correlate for fresh probiotic products (Sielatycka et al., 2021). However, recent studies have compared the performance of flow cytometry during long-term probiotic stability studies to PC enumeration using predictive microbiology (Foglia et al., 2020; Visciglia et al., 2022). The studies revealed that as the storage temperature increases, the CFUs decrease faster than AFUs, suggesting that the loss of cultivability is quicker than the loss of membrane integrity. Yet, the metabolic potential of the probiotic products was maintained, observed as the ability to acidify a fermentation broth and hinting that the product would be able to exhibit its beneficial effects under appropriate circumstances (Visciglia et al., 2022).

The difference between AFU and CFU readings might be attributed to cells that exist in a VBNC state, bacterial populations exhibiting metabolic activity but loss of cultivability (Lotoux et al., 2022). This situation is often observed in probiotic products due to the numerous unavoidable stressful conditions that probiotic cultures undergo during industrial production and the shelf-life of the finished product (Wendel, 2022).

Discrepancies between AFU and CFU counts can also be observed in multi-strain probiotic formulations where several issues may arise and impede PC effectiveness. For one, interactions between the different strains in a blend, such as competition for nutrients or the production of inhibitory compounds, could underestimate the total count of probiotic bacteria determined using PC compared to counts determined using a cytometric method (Sielatycka et al., 2021). Method suitability factors such as growth enhancers, incubation time, incubation temperature, and oxygen conditions (aerobic, microaerophilic, anaerobic) also play a critical role (Sielatycka et al., 2021).

Beyond the ISO 19344 IDF 232 protocol, other fluorescent dyes with alternative properties can be used, such as carboxyfluorescein diacetate succinimide ester (CFDA), which binds to intracellular proteins of intact cells (Ma et al., 2023), or CellROX^®^ Green Reagent, a DNA-binding cell-permeant dye that exhibits bright green fluorescence when oxidized by reactive oxygen species (ROS) (Fallico et al., 2020).

FCM cell counting is based either on a standard reference microsphere counting method or an absolute enumeration (volume method), where the actual number of target cells in a sample is determined using the optical characteristics of the cells and the sample volume. Advances in FCM have further improved volume methods by using acoustic focusing, which generates ultrasonic waves to transport particles to the center of the sample stream, reducing analysis time (Ward and Kaduchak, 2018).

Impedance Flow Cytometry (IFC) is a less well-known but promising label-free, non-invasive technology. It relies on the electrical characteristics of the cell. Since viable bacteria have lipid membranes that resist electricity propagation, IFC uses microfluidic channels where bacteria pass through electric fields one cell at a time. Each bacterium results in a small perturbation, and by analyzing the change in electricity, it’s possible to determine if a cell’s membrane is intact or compromised (Clausen et al., 2018; Bertelsen et al., 2020).

A notable limitation of both ISO 19344 FCM enumeration and PC methodologies is their inability to discriminate different species or strains within a blend. Only a few ISO methods, such as ISO 20128:2006 for *Lactobacillus acidophilus* group and ISO 29981:2010 for *Bifidobacterium* genus, provide selective enumeration of probiotic microorganisms using PC. Hence, enumerating individual strains in a multi-strain blend using either PC or FCM methods remains a significant challenge.

However, there have been some interesting attempts to enumerate and identify multi-strain blends using FCM, notably through the use of strain-specific antibodies. Chiron et al. (2018) managed to produce custom polyclonal antibodies against five commercial probiotic strains, successfully enumerating and differentiating closely related strains within three different probiotic food supplements. Bellais et al. (2022) employed flow cytometry and cell sorting to detect, separate, isolate, and then cultivate novel anaerobic strains from human fecal matter, demonstrating the potential of this approach for handling complex bacterial microbiota. Meanwhile, Yang et al. (2017) developed a polyclonal antiserum against the recombinant pilus protein of *L. rhamnosus* GG strain, which is essential for its adherence to the intestinal epithelium. These studies collectively show the feasibility of developing strain-specific antibodies, even those specific to functional traits like strains’ pili, for identifying and enumerating strains in complex bacterial communities, such as commercial blends.

Furthermore, the complexity and cost of such developments should not be underestimated. The success in obtaining strain specificity, hence the development of antibodies, hinges on checking against antibody cross-reactivity. This is technically possible but conceptually complex as commercial probiotic products can comprise strains from various producers and in different quantities. Therefore, the absence of cross-reactivity should ideally be validated against the largest possible number of different commercial strains. However, this is not realistic as each producer has its cell bank, and not all strains are available from culture collections. Further, the Limit of Quantification (LoQ) and Limit of Detection (LoD) must be validated in a relative abundance experiment. Such validation should answer the question, “Am I able to discriminate and quantify each single strain in a blend with strains from different suppliers and in different quantities?”

It is widely accepted that the FCM Method is not only faster but also more accurate than the PC method. Notably, the interpretation of results from these two methods – Total Fluorescent Units (TFU) and/or Colony Forming Units (CFU) for FCM and PC, respectively, – should be separated from their biological significance and their correlation. It has been reiterated that a close to 1:1 correlation between Active Fluorescent Units (AFU) and CFU data is typically seen in fresh, non-stressed, single-strain probiotic products. However, this correlation diminishes over time and is affected by variables such as temperature, humidity, and the presence of additional strains. Consequently, attempts to correlate FCM and PC are bound to falter under these conditions.

The value of FCM is found in its rich output, providing a comprehensive overview of the heterogeneity within a bacterial population: total cells, dead cells, live cells, and potential Viable But Nonculturable (VBNC) cells. Coupled with PC, it also provides information on cells capable of replicating under specific cultural conditions. CFU data informs only on the sub-population capable of replicating under given experimental conditions, but it provides no information on the VBNC fraction. Many pathogenic microorganisms that are food-borne, such as *Campylobacter jejuni*, *Campylobacter coli*, *Enterococcus faecalis*, *Escherichia coli*, *Helicobacter pylori*, *Salmonella*, *Shigella*, *Vibrio cholerae*, among others, are known to enter VBNC states (Ramamurthy et al., 2014). For this reason, FCM enumeration is now officially recommended for all freshwater analysis in Switzerland for the detection of pathogens (Egli and Kötzsch, 2015; Van Nevel et al., 2017). If VBNC pathogens pose a risk and need to be managed using FCM because they can thrive when they find themselves in a conducive ecosystem, it is not plausible to presume that probiotics, which also have enteric origins, would behave similarly?

Answering these questions propels us into a new perspective, as FCM results can be expressed as the total number of cells present in a product (TFU) and their heterogeneity in compliance with the staining protocol (ISO 19344: membrane integrity, membrane potential, and enzymatic activity) as AFUs.

FCM methods provide the opportunity to also explore postbiotics, specifically when the cell is of interest, and not its degree of “viability.” This is particularly relevant for applications where probiotic microorganisms may not easily or at all survive (for instance in food ingredients that require cooking, beverages such as tea, coffee, sports drinks, and even water, aggressive industrial processes, and product categories like cosmetics). Nevertheless, quantifying the total number of cells present in a given product becomes functionally relevant if the efficacy is associated with the total number of cells (TFUs).

Finally, Live Biotherapeutics are gaining interest and traction with many novel developments, however most of the bacterial candidates are strictly anaerobic and difficult to propagate, as amply demonstrated by Bellali et al. (2019). An interesting use of FCM in novel strain applications belong to the recent Novel Food approval by EFSA of *Akkermansia municiphila,* which has been approved as a novel ingredient based on the data provided in TFUs since it is a pasteurized ingredient (Turck et al., 2021), which functionality resides not on the cellular metabolism but on a specific membrane protein (Plovier et al., 2017).

## Real time PCR based methods

Another culture-independent probiotic enumeration strategy involves DNA-based methods such as Polymerase Chain Reaction (PCR) methods. PCR is a lab technique that amplifies a particular DNA sequence region, creating millions of copies that are easy to detect. This reaction is driven by two primers (short, single-stranded nucleic acid sequences that serve as DNA synthesis starting points), which create the two ends of the sequence to be amplified, and DNA polymerases that build a new DNA strand based on the complementary strand’s information (van Pelt-Verkuil et al., 2008). PCR runs use thermal cycling to heat and cool the DNA, with each cycle composed of three steps: denaturation at around 95°C, which separates the template DNA double helix into two single strands; annealing at roughly 50–65°C, enabling the primers to bind to a complementary template sequence; and extension or elongation at approximately 72°C, allowing the polymerase enzyme to synthesize a new complementary DNA strand. Hence, the number of copies of the target sequence region theoretically doubles after each cycle (Mullis and Faloona, 1987). There are different platforms to conduct PCR, such as conventional end-point PCR, real-time PCR, and digital PCR. Both real-time PCR and digital PCR can be used for probiotic quantification.

In real-time PCR (qPCR), PCR product accumulation after each cycle can be monitored in real-time using fluorescence signals (Holland et al., 1991; Higuchi et al., 1992). The fluorescence intensity increases as the number of DNA copies increases after each qPCR cycle. Once the fluorescence signal crosses a threshold, fluorescence becomes discernible from the background, marking the quantification cycle (Cq). The Cq is the output from a qPCR run and reflects the initial amount of target DNA in a sample. DNA quantification is achieved by constructing a calibration curve using the initial target DNA amounts and the corresponding Cq values (Kralik and Ricchi, 2017). Fluorescence signals can be measured using non-specific fluorescent DNA dyes such as SYBR Green I or a fluorescently labeled oligonucleotide probe (hydrolysis probe) (Holland et al., 1991; Higuchi et al., 1992; Wilhelm and Pingoud, 2003). The hydrolysis probe chemistry enhances specificity and enables simultaneous detection of multiple targets in one PCR reaction (multiplexing) when multiple primer pairs and a combination of probes with different fluorophores are used (Elnifro et al., 2000).

Real-time PCR methods are targeted methods that can identify specific analytes, such as a particular probiotic species or strain. The capacity to detect a specific species or strain depends on the primers and hydrolysis probe used. Carefully designed primers can identify minimal genetic variations between strains, like single nucleotide polymorphisms. High-quality genome sequences and bioinformatic tools are required to design species- or strain-specific primers and probes. This process can be especially challenging for very closely related targets, like different strains of *Bifidobacterium animalis* subsp. *lactis* (Milani et al., 2013). Notably, strain-specific assays are designed based on sequences available in public databases such as GenBank at the time of assay design. Thus, frequent updates in sequence databases with new sequence deposits may impact the specificity of strain-specific assays and may require designing new methods or modifying existing methods by targeting additional sequence regions to ensure strain level specificity.

Real-time PCR methods have been developed for probiotic strain-specific identification, such as *L. rhamnosus* GG (Ahlroos and Tynkkynen, 2009; Shehata and Newmaster, 2020), *B. animalis* subsp. *lactis* Bb12 (Solano-Aguilar et al., 2008), *B. animalis* subsp. *lactis* DSM 15954 and Bi-07™ (Shehata et al., 2021a), *L. gasseri* BNR17, and *L. reuteri* LRC (NCIMB 30242) (Shehata et al., 2021b).

**Figure 3:**
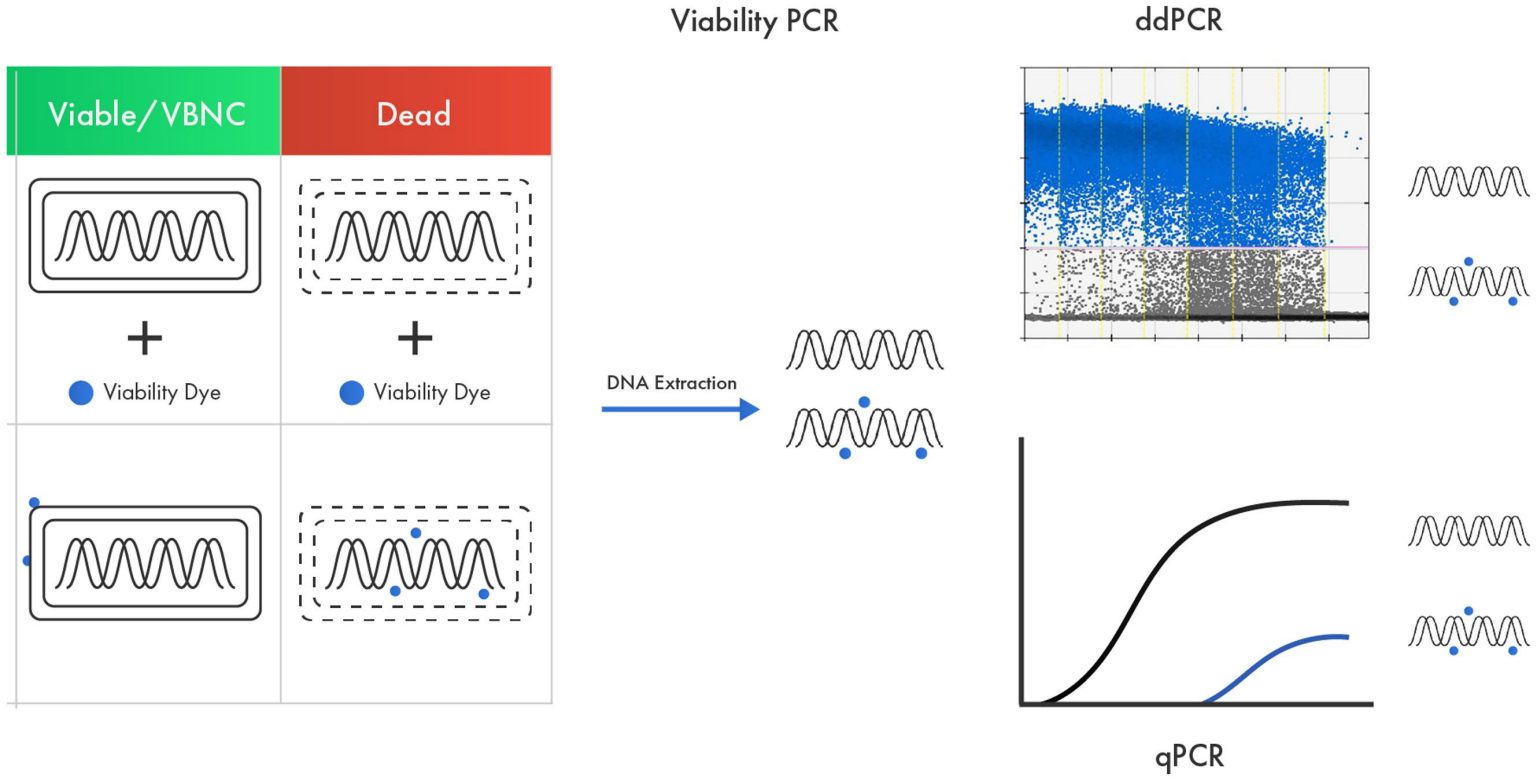
v-qPCR as an advanced tool in probiotic viability assessment based on membrane integrity. A viability dye that can enter only dead cells and membrane-damaged cells and intercalate with their DNA is used to render DNA from dead cells unresponsive in a PCR.

Real-time PCR methods can also offer species-specific or strain-specific enumeration of probiotics (Furet et al., 2004; Achilleos and Berthier, 2013; Herbel et al., 2013). To count only viable cells, viability qPCR (v-qPCR) is used, in which probiotic cells are pretreated with a viability dye like ethidium monoazide (EMA), propidium monoazide (PMA), or modified forms of PMA (Figure 3). These viability dyes render DNA from dead cells unresponsive in a PCR reaction, achieved by their ability to enter dead and membrane-damaged cells and intercalate with their DNA (Fittipaldi et al., 2012; Shehata and Newmaster, 2021). The viability dye treatment must be optimized for each target strain because the effectiveness in inactivating DNA from dead cells differs among targets (Kiefer et al., 2020). After viability dye treatment, bead beating is typically used for DNA liberation, as commercial DNA purification kits do not yield 100% DNA recovery (Mumy and Findlay, 2004; Hansen et al., 2018), and this loss in DNA recovery can lead to an underestimation of the target quantity (Kralik and Ricchi, 2017). Viability qPCR-based methods include methods for enumerating *L. acidophilus* LA-5 and *B. animalis* subsp. *lactis* BB-12 (Kramer et al., 2009; Dias et al., 2020), *Lactococcus* sp., *L. helveticus*, *L. rhamnosus*, and *B. animalis* subsp. *lactis* (Desfossés-Foucault et al., 2012), *L. plantarum* 564 and *L. paracasei* Z-8 (Radulović et al., 2012), *B. bifidum* BF-1 (Fujimoto and Watanabe, 2013), *L. paracasei* (Scariot et al., 2018), *L. rhamnosus* GG (Shehata and Newmaster, 2021), and *L. paracasei* 8,700:2 (Shehata et al., 2023).

Several studies have compared how v-qPCR counts align with PC, the standard enumeration method. Most studies found an agreement between counts determined using both methods. For instance, PC and v-PCR methods yielded relatively similar results when quantifying *L. acidophilus* LA-5 and *B. animalis* ssp. *lactis* BB-12 in lyophilised products (Kramer et al., 2009). In another study, bacterial counts of spray-dried *L. plantarum* 564 and *L. paracasei* Z-8 determined using v-PCR were not significantly different from results determined using PC methods (Radulović et al., 2012). Scariot et al. (2018) found that plate counts were comparable to v-PCR counts for *L. paracasei* viable cells in yogurt. Another study reported similar cell counts for *B. animalis* subsp. *lactis* by v-qPCR and PC on a selective media during 30 days of storage at 4°C (Dias et al., 2020). On the other hand, the counts of *B. bifidum* BF-1 determined by v-PCR method was approximately 50 times higher than plate counts on selective agar supplemented with antibiotics, which was attributed to the use of antibiotics leading to underestimation of viable cells (Fujimoto and Watanabe, 2013). The viable counts of *L. paracasei* 8,700:2 by v-qPCR were higher than the PC method, which may be attributed to cells in a VBNC state (Shehata et al., 2023).

Despite the numerous benefits of v-qPCR, it is important to acknowledge its limitations, as well as the workarounds that have been developed to address them. One inherent challenge in the v-qPCR methodology is the necessity to design specific primers and probes for each target species or strain to be quantified. The level of bioinformatic analysis required can increase substantially when the target strain is highly genetically similar to other strains, making precise identification more difficult.

Moreover, every v-qPCR method needs to be meticulously optimized and validated for several key parameters: specificity, sensitivity, repeatability, reproducibility, and practicability (Bustin et al., 2009; Broeders et al., 2014). This includes optimizing the viability dye treatment to ensure the detection of live cells. If not thoroughly validated, the v-qPCR method will not yield accurate quantification results. For instance, an assay that is not fully specific to the target could lead to an overestimation of the quantity, as it may inadvertently pick up other targets present in the test sample. Likewise, an assay with a reaction efficiency outside the ideal range of 90–110% could either underestimate or overestimate the target quantity.

Additionally, each assay must be validated for various sample matrices to assess their performance and confirm the absence of inhibitory effects from other components in the sample. Despite these challenges, robust assay design and comprehensive validation can effectively mitigate these limitations, enabling reliable results.

Nevertheless, v-qPCR remains a compelling choice for probiotic enumeration due to its distinct advantages over traditional culture-based methods. These benefits include higher precision, higher throughput, and a significantly shorter time to results (approximately 10 times faster), and the ability to achieve quantifications that are not possible with culture-based methods. For instance, v-qPCR can enumerate individual strains in multi-strain blends (Jackson et al., 2019; Shehata and Newmaster, 2021; Shehata et al., 2023), which is particularly valuable when evaluating product stability during shelf life. Furthermore, v-qPCR methods can quantify viable but non-culturable (VBNC) cells (Kell et al., 1998; Lahtinen et al., 2006a; Davis, 2014; Wilkinson, 2018; Gorsuch et al., 2019), NGPs and potentially some types of postbiotics. For example, v-qPCR is applicable to heat-killed cells where DNA is expected to be present, but not applicable to purified components or metabolites. Therefore, despite the complexity of the optimization and validation processes, v-qPCR offers promising potential for comprehensive and efficient probiotic enumeration.

## Digital PCR based methods

Digital PCR represents a powerful technique for probiotic enumeration, building on the core principles of real-time PCR, and includes several distinct characteristics (Table 2). Like real-time PCR, digital PCR uses species-specific- or strain-specific primers along with fluorescent dyes or probes to amplify and identify specific genomic regions. What sets digital PCR apart is the unique approach it takes: it distributes the target molecules individually into many small partitions and runs PCR on each single molecule across thousands of simultaneous reactions (Vogelstein and Kinzler, 1999). This yields a positive fluorescent signal for each positive reaction, which a fluorimeter then reads. By applying Poisson’s law of small numbers, the ratio of positive to negative signals can be calculated, thereby producing a quantitative value, typically in copies per microliter (Jacobs et al., 2017).

**Table 2:** Comparing real-time PCR and digital PCR for probiotic enumeration.

	Real-time PCR	Digital PCR
Taxonomic resolution	Strain-specific or species-specific	Strain-specific or species-specific
Primer design	Required	Required
Robust validation	Required	Required
Multiplexing	Yes	Yes
Throughput	Up to 384-well	Up to 96-well
Calibration curve	Required	Not required
Inhibitors	Prone to inhibitors	High tolerance to inhibitors
Effect of PCR efficiency on results	Results are affected by PCR efficiency	Results are not affected by PCR efficiency
Real-time monitoring	Yes	No
Dynamic range	Broad dynamic range	Narrower dynamic range
Equipment and running costs	Economical	Less economical

Different partitioning technologies have been developed for digital PCR, with chip-based systems (cdPCR), plate-based systems, and oil droplet-based systems (ddPCR) being among the most common. In chip-based platforms, microfluidics are used to partition individual molecules into thousands of microscopic wells on a chip or plate. These platforms then perform end-point PCR detection with fluorescence on the chip (Zhang and Xing, 2007; Sanders et al., 2011). Plate-based platforms, meanwhile, are scalable, with plates housing up to 96 individual wells partitioned in a manner similar to chip-based systems. Droplet-based platforms create thousands of microscopic droplets using a droplet generator in a process involving an immiscible fluid in oil. The target nucleic acid is randomly encapsulated in the droplets, and end-point PCR is then performed. Positive signals are processed and analyzed using Poisson statistics, yielding an absolute count of the DNA copies present (Gobert et al., 2018). The evolution of instrumentation has facilitated the ability to multiplex up to five targets in a single reaction.

Similar to real-time PCR, the digital PCR method requires thorough optimization and validation to ensure specificity, sensitivity, repeatability, reproducibility, and practicability of the reaction (Broeders et al., 2014). ddPCR has shown greater sensitivity than real-time PCR in detecting low bacterial concentrations in dairy products spiked with bacteria (Kim et al., 2023). Additionally, digital PCR has demonstrated a higher tolerance to PCR inhibitors, which makes it a preferred choice for detecting low levels of target organisms in complex matrices like soil and wastewater (Rački et al., 2014).

Studies comparing the performance characteristics of real-time PCR and digital PCR have shown good linearity and high coefficients of determination for both platforms when quantifying *Lactiplantibacillus plantarum* subsp. *plantarum* in raw material and food matrices. Digital PCR displayed a 10-fold lower limit of detection, suggesting superior sensitivity, but demonstrated limitations when quantifying high probiotic concentrations (Choi et al., 2022). Comparative analyses of v-qPCR and v-ddPCR on *L. paracasei* 8700.2 revealed very high correlation and no significant differences (Shehata et al., 2023).

One key advantage of dPCR over qPCR in probiotic enumeration is that the results generated are not influenced by reaction efficiency or standard curve calibration, leading to enhanced precision (Hindson et al., 2013; Raurich et al., 2019). Several studies have reported improved accuracy and reproducibility with dPCR compared to qPCR (Pinheiro et al., 2012; Nshimyimana et al., 2019). The advantages of v-qPCR, including better precision, reduced labor, higher throughput, species- or strain-specific enumeration based on primer specificity, and the ability to detect and quantify VBNC states, can be directly translated to dPCR as both techniques share similar principles.

However, some of the same limitations apply to dPCR as well, such as the need for primer specificity, which entails comprehensive bioinformatic analysis. Each method must be individually optimized and thoroughly validated to ensure confidence in the results produced. Despite these challenges, the promise of dPCR for reliable and accurate enumeration of probiotics continues to generate interest.

## Culture-independent methods in the probiotic industry

New culture-independent methods are proving to be particularly beneficial in research and development stages of product design, especially in experimental settings where a multitude of microorganisms are examined for specific functions (Supplementary Table S1). Clinical studies play a pivotal role in demonstrating the efficacy of probiotics, and accurate probiotic cell count, or concentration, is fundamental to correlate the health benefit with the delivered dose. However, only about 42% of global clinical trials on probiotics accurately reported the dosage of the tested products, generally in CFUs (Dronkers et al., 2020). Furthermore, most studies do not specify the point at which the product concentration is measured: at manufacturing (Quantity by Input), point of consumption, or end of shelf life (Goldman, 2019).

Unfortunately, CFU count is inherently flawed when it comes to standardization and comparison between various biological isolates and experiments. CFUs provide insight into a potentially viable subgroup of micro-organisms capable of forming a colony, but do not offer a complete picture of the entire bacterial population within a sample. It’s important to consider that humans consume the entire spectrum of bacterial population heterogeneity (Fiore et al., 2020). This varied heterogeneity of a given probiotic or potential probiotic may contribute to diverse functional characteristics. This raises compelling questions: Should consumers be informed about this complexity?

Furthermore, the post-experimental, not real-time, correlation with CFU makes comprehensive analysis and comparison of published literature rather complex (Davey, 2011). Even at the industrial production level, real-time monitoring of cell number and heterogeneity can fine-tune the process, aiming for the highest possible viability in the finished product (Supplementary Table S1). However, achieving a one-to-one correlation between plate count (CFUs) and other viability proxies is challenging and unlikely, especially when the product is composed of multiple microorganisms, each with its specific characteristics and industrial process, or when monitored for its shelf-life (Foglia et al., 2020; Visciglia et al., 2022).

From a commercial perspective, the insistence on strain-level identity poses challenges for companies formulating probiotic products, especially when dealing with multi-strain blends or complex formulations. Ensuring that each strain included in a product is individually characterized and that their combined effects have been clinically validated can be a daunting and costly task. From this perspective, it seems easier to formulate products with a single strain or a few easily identifiable strains. The validation process would realistically be more suited to the producers rather than the Contract Manufacturing Organization (CMO), which potentially formulates sourcing from different producers.

The ever-evolving probiotic market and growing interest from regulators and large companies put pressure on the need for more robust quality controls. However, lack of harmonized regulations and market diversification results in a multitude of products, with the only quality information often being the label details. In a future scenario where strain discrimination and enumeration become mandatory, considerations must be given to smaller companies with limited resources and contract manufacturing organizations (CMOs) that primarily work with blends of various strains. In fact, with a requirement to demonstrate the qualitative-quantitative composition at the end of the shelf-life, there might be a progressive move away from multi-strain products toward simpler formulas. A balanced approach might be to promote species level identification and enumeration while encouraging the achievement of strain specificity. The key is to consider product design within the available methodologies.

Culture-independent methods also offer alternative solutions for enumerating heat-killed bacteria, which can provide significant insights into the process of tyndallization and its potential improvements to achieve higher yields without damaging cells (Supplementary Table S1). By using dyes to differentiate live from dead cells based on membrane integrity, these methods allow for a more accurate yet rapid approach similar to the counting chamber.

Culture-independent methods have the potential to facilitate market access, especially for hot climate zones (Zone IVa and Zone IVb with 30°C and 65 and 75% Relative Humidity (RH) respectively) such as Asia, India, Latin America, and North Africa. To ensure the guaranteed concentration at the end of the product’s shelf life, manufacturers often increase the initial concentration two to three-fold, or even higher. This increase, often referred to as “overage,” is typically determined through stability studies conducted on the final product under recommended storage conditions (Roe et al., 2022). In fact, designing products with a CFU target at the end of the shelf-life compared to alternative methods based on membrane integrity implies higher overages than in temperate markets (Foglia et al., 2020; Visciglia et al., 2022). These overages are inherently limited by factors such as space, homogeneity, etc., and notably, price – a factor that significantly impacts market access in less affluent countries.

It appears that rather than debating the correlation between methods, especially the ones that probe viability by different means, the focus should be on improving the procedures to track and report experimental and clinical data. Enhancing these procedures and providing regulatory framing for them is critical. This approach would ensure that accurate, reliable data is available for all stakeholders in the probiotic industry. Harmonizing these procedures globally could also provide a standard against which all probiotic products are measured, enhancing the probiotic industry’s credibility and fostering trust in these products’ efficacy and safety.

## Conclusion

In conclusion, the field of probiotics research and production has seen remarkable advances over the past decades. However, challenges still exist in the methods used for quantifying and characterizing these beneficial microorganisms. Traditional culture-dependent methods, such as CFU enumerations and optical density measurements, lack the precision and the comprehensiveness required for standardization and comparison across various studies and strains. Culture-independent methods, including flow cytometry and PCR-based techniques, have emerged as promising alternatives that provide real-time, strain-specific data and offer a deeper understanding of the heterogeneity and viability of bacterial populations. The principles of culture-independent methods align with the official probiotic definition (Hill et al., 2014), defined as: “live microorganisms that, when administered in adequate amounts, confer a health benefit on the host.,” as they produce outputs indicative of cellular viability.

These methods, however, are not without their challenges, particularly when it comes to the development of strain-specific markers, antibodies for flow cytometry and primers and probes for qPCR and dPCR. Creating a central depository for commercial strains, physical materials and whole genome sequences, would be of great benefit when evaluating the strain specificity of the developed strain-specific markers. As the probiotics field continues to mature, it is critical that the scientific community and industry stakeholders work collaboratively to further refine these methods, champion their adoption, and work toward the establishment of global, harmonized standards. This will not only enhance the reproducibility and comparability of research data, but also ensure the delivery of high-quality, well-characterized probiotic products to consumers, underpinning their confidence in the market and driving the growth of this important sector. The advancement and refinement of these techniques have potential implications far beyond the probiotics field, heralding a new era in microbial research and its numerous applications across various domains of human health.

## Author contributions

M-EB: Conceptualization, Methodology, Project administration, Writing – original draft, Writing – review & editing. AB: Conceptualization, Methodology, Writing – original draft, Writing – review & editing. MP: Conceptualization, Methodology, Writing – original draft, Writing – review & editing. HS: Conceptualization, Methodology, Writing – original draft, Writing – review & editing.
